# Supplementary material for: COZOID: contact zone identifier for visual analysis of protein-protein interactions
Source: BMC Bioinformatics. 2018 Apr 6;19:125. doi: 10.1186/s12859-018-2113-6 (PMC5889581; doi:10.1186/s12859-018-2113-6)
Supplement: Supplementary file 2 — Software build. Executable binary file of the software tool. (ZIP 118,784 kb) [file 12859_2018_2113_MOESM2_ESM.zip › cozoid/java/docs/beansbinding-1.2.1-doc.zip!/index-files/index-19.html]

S-Index (Beans Binding)


---


|  |  |  |
| --- | --- | --- |
| **Overview** \| Package \| Class \| Use \| **Tree** \| **Deprecated** \| **Index** \| **Help** | |  |
| **PREV LETTER**   **NEXT LETTER** | **FRAMES**    **NO FRAMES**     **All Classes** |  |


A B C D E F G H I J K L M N O P Q R S T U V W 

---


## **S**

**save()** - Method in class org.jdesktop.beansbinding.Binding: Fetches the value of the target property for the target object and sets it as the value of the source property for the source object. **saveAndNotify()** - Method in class org.jdesktop.beansbinding.Binding: The same as `Binding.save()` with the additional behavior of notifying all registered `BindingListeners` with `synced` if `save` returns `null` or `syncFailed` if `save` returns a `SyncFailure`. **saveAndNotifyUnmanaged()** - Method in class org.jdesktop.beansbinding.Binding: A protected version of `Binding.saveAndNotify()` that allows managed subclasses to save and notify without throwing an exception for being managed. **saveUnmanaged()** - Method in class org.jdesktop.beansbinding.Binding: A protected version of `Binding.save()` that allows managed subclasses to save without throwing an exception for being managed. **setColumn(int)** - Method in class org.jdesktop.swingbinding.impl.AbstractColumnBinding: **setColumnClass(Class<?>)** - Method in class org.jdesktop.swingbinding.JTableBinding.ColumnBinding: Sets the column class to be used by `JTable` to determine the renderer and editor for the column represented by this `ColumnBinding`. **setColumnName(String)** - Method in class org.jdesktop.swingbinding.JTableBinding.ColumnBinding: Sets a name for the column represented by this `ColumnBinding`. **setConverter(Converter<SV, TV>)** - Method in class org.jdesktop.beansbinding.Binding: Sets the `Converter` for the `Binding`, which may be `null`. **setDebugStream(PrintStream)** - Method in class org.jdesktop.el.impl.parser.ELParserTokenManager: **setDetailBinding(Property<E, ?>)** - Method in class org.jdesktop.swingbinding.JListBinding: Creates a `DetailBinding` and sets it as the `DetailBinding` for this `JListBinding`. **setDetailBinding(Property<E, ?>, String)** - Method in class org.jdesktop.swingbinding.JListBinding: Creates a named `DetailBinding` and sets it as the `DetailBinding` for this `JListBinding`. **setEditable(boolean)** - Method in class org.jdesktop.swingbinding.JTableBinding.ColumnBinding: Sets whether or not the cells of the column should be editable. **setEditable(boolean)** - Method in class org.jdesktop.swingbinding.JTableBinding: Sets whether or not the cells of the table should be editable. **setElements(List<?>, boolean)** - Method in class org.jdesktop.swingbinding.impl.ListBindingManager: **setImage(String)** - Method in class org.jdesktop.el.impl.parser.AstLiteralExpression: **setImage(String)** - Method in class org.jdesktop.el.impl.parser.AstString: **setImage(String)** - Method in class org.jdesktop.el.impl.parser.SimpleNode: **setLocale(Locale)** - Method in class org.jdesktop.el.ELContext: Set the `Locale` for this instance. **setLocalName(String)** - Method in class org.jdesktop.el.impl.parser.AstFunction: **setManaged(boolean)** - Method in class org.jdesktop.beansbinding.Binding: Sets whether or not this `Binding` is managed. **setPrefix(String)** - Method in class org.jdesktop.el.impl.parser.AstFunction: **setPropertyResolved(boolean)** - Method in class org.jdesktop.el.ELContext: Called to indicate that a `ELResolver` has successfully resolved a given (base, property) pair. **setPropertyResolved(boolean)** - Method in class org.jdesktop.el.impl.lang.EvaluationContext: **setSelected(boolean)** - Method in class org.jdesktop.swingbinding.adapters.AbstractButtonAdapterProvider.Adapter: **setSelectedItem(Object)** - Method in class org.jdesktop.swingbinding.adapters.JComboBoxAdapterProvider.Adapter: **setSource(Object)** - Method in class org.jdesktop.el.ValueExpression: Sets the source of the expression. **setSourceNullValue(TV)** - Method in class org.jdesktop.beansbinding.Binding: Sets the value to be returned by `Binding.getSourceValueForTarget()` when the source property returns `null` for the source object. **setSourceObject(SS)** - Method in class org.jdesktop.beansbinding.Binding: Sets the `Binding's` source object, which may be `null`. **setSourceObjectUnmanaged(SS)** - Method in class org.jdesktop.beansbinding.Binding: A protected version of `Binding.setSourceObject(SS)` that allows managed subclasses to set the source object without throwing an exception for being managed. **setSourceProperty(Property<SS, SV>)** - Method in class org.jdesktop.beansbinding.Binding: Sets the `Binding's` source property. **setSourceUnreadableValue(TV)** - Method in class org.jdesktop.beansbinding.Binding: Sets the value to be returned by `Binding.getSourceValueForTarget()` when the source property is unreadable for the source object. **setTargetNullValue(SV)** - Method in class org.jdesktop.beansbinding.Binding: Sets the value to be returned by `Binding.getTargetValueForSource()` when the target property returns `null` for the target object. **setTargetObject(TS)** - Method in class org.jdesktop.beansbinding.Binding: Sets the `Binding's` target object, which may be `null`. **setTargetObjectUnmanaged(TS)** - Method in class org.jdesktop.beansbinding.Binding: A protected version of `Binding.setTargetObject(TS)` that allows managed subclasses to set the target object without throwing an exception for being managed. **setTargetProperty(Property<TS, TV>)** - Method in class org.jdesktop.beansbinding.Binding: Sets the `Binding's` target property. **setText(String)** - Method in class org.jdesktop.swingbinding.adapters.JTextComponentAdapterProvider.Adapter: **setText\_ON\_ACTION\_OR\_FOCUS\_LOST(String)** - Method in class org.jdesktop.swingbinding.adapters.JTextComponentAdapterProvider.Adapter: **setText\_ON\_FOCUS\_LOST(String)** - Method in class org.jdesktop.swingbinding.adapters.JTextComponentAdapterProvider.Adapter: **setValidator(Validator<? super SV>)** - Method in class org.jdesktop.beansbinding.Binding: Sets the `Validator` for the `Binding`, which may be `null`. **setValue(S, V)** - Method in class org.jdesktop.beansbinding.BeanProperty: Sets the value of this `Property` for the given source. **setValue(S, V)** - Method in class org.jdesktop.beansbinding.ELProperty: Sets the value of this `Property` for the given source. **setValue(S, S)** - Method in class org.jdesktop.beansbinding.ObjectProperty: Throws `UnsupportedOperationException`; `ObjectProperty` is never writeable. **setValue(S, V)** - Method in class org.jdesktop.beansbinding.Property: Sets the value of this `Property` for the given source. **setValue(S, V)** - Method in class org.jdesktop.beansbinding.PropertyHelper: Sets the value of this `Property` for the given source. **setValue(ELContext, Object, Object, Object)** - Method in class org.jdesktop.el.ArrayELResolver: If the base object is a Java language array, attempts to set the value at the given index with the given value. **setValue(ELContext, Object, Object, Object)** - Method in class org.jdesktop.el.BeanELResolver: If the base object is not `null`, attempts to set the value of the given property on this bean. **setValue(ELContext, Object, Object, Object)** - Method in class org.jdesktop.el.CompositeELResolver: Attempts to set the value of the given `property` object on the given `base` object. **setValue(ELContext, Object, Object, Object)** - Method in class org.jdesktop.el.ELResolver: Attempts to set the value of the given `property` object on the given `base` object. **setValue(EvaluationContext, Object)** - Method in class org.jdesktop.el.impl.parser.AstDeferredExpression: **setValue(EvaluationContext, Object)** - Method in class org.jdesktop.el.impl.parser.AstDynamicExpression: **setValue(EvaluationContext, Object)** - Method in class org.jdesktop.el.impl.parser.AstIdentifier: **setValue(EvaluationContext, Object)** - Method in class org.jdesktop.el.impl.parser.AstValue: **setValue(EvaluationContext, Object)** - Method in interface org.jdesktop.el.impl.parser.Node: **setValue(EvaluationContext, Object)** - Method in class org.jdesktop.el.impl.parser.SimpleNode: **setValue(ELContext, Object)** - Method in class org.jdesktop.el.impl.ValueExpressionImpl: **setValue(ELContext, Object)** - Method in class org.jdesktop.el.impl.ValueExpressionLiteral: **setValue(ELContext, Object, Object, Object)** - Method in class org.jdesktop.el.ListELResolver: If the base object is a list, attempts to set the value at the given index with the given value. **setValue(ELContext, Object, Object, Object)** - Method in class org.jdesktop.el.MapELResolver: If the base object is a map, attempts to set the value associated with the given key, as specified by the `property` argument. **setValue(ELContext, Object, Object, Object)** - Method in class org.jdesktop.el.ResourceBundleELResolver: If the base object is a ResourceBundle, throw a `PropertyNotWritableException`. **setValue(ELContext, Object)** - Method in class org.jdesktop.el.ValueExpression: Evaluates the expression relative to the provided context, and sets the result to the provided value. **setValue(int)** - Method in class org.jdesktop.swingbinding.adapters.JSliderAdapterProvider.Adapter: **setValue(Object)** - Method in class org.jdesktop.swingbinding.adapters.JSpinnerAdapterProvider.Adapter: **setValue\_IGNORE\_ADJUSTING(int)** - Method in class org.jdesktop.swingbinding.adapters.JSliderAdapterProvider.Adapter: **setVariable(String, ValueExpression)** - Method in class org.jdesktop.el.impl.lang.VariableMapperFactory: **setVariable(String, ValueExpression)** - Method in class org.jdesktop.el.impl.lang.VariableMapperImpl: **setVariable(String, ValueExpression)** - Method in class org.jdesktop.el.VariableMapper: Assign a ValueExpression to an EL variable, replacing any previously assignment to the same variable. **SimpleCharStream** - Class in org.jdesktop.el.impl.parser: An implementation of interface CharStream, where the stream is assumed to contain only ASCII characters (without unicode processing). **SimpleCharStream(Reader, int, int, int)** - Constructor for class org.jdesktop.el.impl.parser.SimpleCharStream: **SimpleCharStream(Reader, int, int)** - Constructor for class org.jdesktop.el.impl.parser.SimpleCharStream: **SimpleCharStream(Reader)** - Constructor for class org.jdesktop.el.impl.parser.SimpleCharStream: **SimpleCharStream(InputStream, int, int, int)** - Constructor for class org.jdesktop.el.impl.parser.SimpleCharStream: **SimpleCharStream(InputStream, int, int)** - Constructor for class org.jdesktop.el.impl.parser.SimpleCharStream: **SimpleCharStream(InputStream)** - Constructor for class org.jdesktop.el.impl.parser.SimpleCharStream: **SimpleNode** - Class in org.jdesktop.el.impl.parser: **SimpleNode(int)** - Constructor for class org.jdesktop.el.impl.parser.SimpleNode: **size()** - Method in class org.jdesktop.swingbinding.impl.ListBindingManager: **sourceChanged(Binding, PropertyStateEvent)** - Method in class org.jdesktop.beansbinding.AbstractBindingListener: Notification that the source property of a `Binding` has fired a `PropertyStateEvent` for the `Binding's` source object. **sourceChanged(Binding, PropertyStateEvent)** - Method in interface org.jdesktop.beansbinding.BindingListener: Notification that the source property of a `Binding` has fired a `PropertyStateEvent` for the `Binding's` source object. **sourceChangedImpl(PropertyStateEvent)** - Method in class org.jdesktop.beansbinding.AutoBinding: **sourceChangedImpl(PropertyStateEvent)** - Method in class org.jdesktop.beansbinding.Binding: Called to indicate that the source property has fired a `PropertyStateEvent` to indicate that its state has changed for the source object. **sourceEdited(Binding)** - Method in class org.jdesktop.beansbinding.AbstractBindingListener: **Deprecated.** *This method has been replaced by `AbstractBindingListener.sourceChanged(org.jdesktop.beansbinding.Binding, org.jdesktop.beansbinding.PropertyStateEvent)` and it will go away soon. It is being kept for a short period only, to assist in migration.* **specialConstructor** - Variable in exception org.jdesktop.el.impl.parser.ParseException: This variable determines which constructor was used to create this object and thereby affects the semantics of the "getMessage" method (see below). **specialToken** - Variable in class org.jdesktop.el.impl.parser.Token: This field is used to access special tokens that occur prior to this token, but after the immediately preceding regular (non-special) token. **START\_DEFERRED\_EXPRESSION** - Static variable in interface org.jdesktop.el.impl.parser.ELParserConstants: **START\_DYNAMIC\_EXPRESSION** - Static variable in interface org.jdesktop.el.impl.parser.ELParserConstants: **staticFlag** - Static variable in class org.jdesktop.el.impl.parser.SimpleCharStream: **String()** - Method in class org.jdesktop.el.impl.parser.ELParser: **STRING\_LITERAL** - Static variable in interface org.jdesktop.el.impl.parser.ELParserConstants: **subtract(Number, Number)** - Method in class org.jdesktop.el.impl.lang.ELArithmetic.BigDecimalDelegate: **subtract(Number, Number)** - Method in class org.jdesktop.el.impl.lang.ELArithmetic.BigIntegerDelegate: **subtract(Number, Number)** - Method in class org.jdesktop.el.impl.lang.ELArithmetic.DoubleDelegate: **subtract(Number, Number)** - Method in class org.jdesktop.el.impl.lang.ELArithmetic.LongDelegate: **subtract(Object, Object)** - Static method in class org.jdesktop.el.impl.lang.ELArithmetic: **subtract(Number, Number)** - Method in class org.jdesktop.el.impl.lang.ELArithmetic: **supportsElementPropertyChanged()** - Method in interface org.jdesktop.observablecollections.ObservableList: Returns `true` if this list sends out notification when the properties of an element change. **SwingBindings** - Class in org.jdesktop.swingbinding: A factory class for creating instances of the custom Swing `Binding` implementations provided by this package. **SwitchTo(int)** - Method in class org.jdesktop.el.impl.parser.ELParserTokenManager: **synced(Binding)** - Method in class org.jdesktop.beansbinding.AbstractBindingListener: Notification that the source and target of a `Binding` have been made in sync. **synced(Binding)** - Method in interface org.jdesktop.beansbinding.BindingListener: Notification that the source and target of a `Binding` have been made in sync. **syncFailed(Binding, Binding.SyncFailure)** - Method in class org.jdesktop.beansbinding.AbstractBindingListener: Notification that the `Binding` attempted to sync the source and target, but the sync failed. **syncFailed(Binding, Binding.SyncFailure)** - Method in interface org.jdesktop.beansbinding.BindingListener: Notification that the `Binding` attempted to sync the source and target, but the sync failed.

---


|  |  |  |
| --- | --- | --- |
| **Overview** \| Package \| Class \| Use \| **Tree** \| **Deprecated** \| **Index** \| **Help** | |  |
| **PREV LETTER**   **NEXT LETTER** | **FRAMES**    **NO FRAMES**     **All Classes** |  |


A B C D E F G H I J K L M N O P Q R S T U V W 

---
